# Clinical diagnostic application of metagenomic next-generation sequencing in children with severe nonresponding pneumonia

**DOI:** 10.1371/journal.pone.0232610

**Published:** 2020-06-04

**Authors:** Heping Wang, Zhiwei Lu, Yaomin Bao, Yonghong Yang, Ronald de Groot, Wenkui Dai, Marien I. de Jonge, Yuejie Zheng

**Affiliations:** 1 Department of Respiratory Diseases, Shenzhen Children’s Hospital, Shenzhen, China; 2 Section Pediatric Infectious Diseases, Laboratory of Medical Immunology, Radboud Center for Infectious Diseases, Radboud university medical center, Nijmegen, The Netherlands; 3 Department of Computer Science, City University of Hong Kong, Hong Kong, China; University of Lincoln, UNITED KINGDOM

## Abstract

Pneumonia is one of the most important causes of morbidity and mortality in children. Identification and characterization of pathogens that cause infections are crucial for accurate treatment and accelerated recovery. However, in most cases, the causative agent cannot be identified, which is partly due to the limited spectrum of pathogens covered by current diagnostics based on nucleic acid amplification. Therefore, in this study, we explored the application of metagenomic next-generation sequencing (mNGS) for the diagnosis of children with severe pneumonia. From April to July 2017, 32 hospitalized children with severe nonresponding pneumonia in Shenzhen Children’s Hospital were included in this study. Blood tests were conducted immediately after hospitalization to assess cell counts and inflammatory markers, oropharyngeal swabs were collected to identify common pathogens by qPCR and culture. After bronchoscopy, bronchoalveolar lavage fluid (BALF) samples were collected for further pathogen identification using standardized diagnostic tests and mNGS. Blood tests were normal in 3 of the 32 children. In 9 oropharyngeal swabs, bacterial pathogens were detected, in 5 of these *Mycoplasma pneumoniae* was detected. Adenovirus was detected in 5 BALF samples, using the Direct Immunofluorescence Assay (DFA). In 15 cases, no common pathogens were found in BALF samples, using the current standard diagnostic tests, while in all 32 BALFs, pathogens were identified using mNGS, including adenovirus, *Mycoplasma pneumoniae*, *Streptococcus pneumoniae*, *Haemophilus influenzae*, *Moraxella catarrhalis*, cytomegalovirus and bocavirus. This study shows that, with mNGS, the sensitivity of detection of the causative pathogens in children with severe nonresponding pneumonia is significantly improved. In addition, mNGS gives more strain specific information, helps to identify new pathogens and could potentially help to trace and control outbreaks. In this study, we have shown that it is possible to have the results within 24 hours, making the application of mNGS feasible for clinical diagnostics.

## Introduction

Pneumonia is one of the most important causes of morbidity and mortality in children [1]. A multitude of pathogens have been identified as potential causes, making it challenging to determine the microbial etiology of pneumonia. Identification and characterization of micro-organisms that cause infections are crucial for targeted treatment, to enable fast recovery of the patients. Culture-based techniques, nucleic acid amplification tests (NAATs) and immunological assays target only a fraction of the currently known pathogens [2]. In addition, culture-based tests require two days and even longer identify the causative pathogen, while immunological assays lack sensitivity and are therefore generally prone to false negative results. This often leads to empirical treatment, solely based on clinical examination, potentially resulting in misuse or overuse of antibiotics.

Metagenomic next-generation sequencing (mNGS) has previously been shown to be a promising technology for the identification of the causative agent in a given sample [3]. The advent of rapid and low-cost mNGS has improved their applications from laboratory research to clinical diagnostics [4–7]. Metagenomic next-generation sequencing has been mainly used to diagnose emerging pathogens and rare infectious diseases [8, 9]. In this study, clinical examinations were conducted and different pathogen detection methods were used simultaneously including mNGS, bacterial culture, *M*. *pneumoniae* PCR and D3 Ultra DFA Respiratory Virus Screening [10] on bronchoalveolar lavage fluid (BALF) samples, to compare differences in diagnostic outcome, to finally adjust the treatment option and monitor the recovery of the patients.

## Methods

### Patient enrollment, clinical assessment and sample collection

From April to July 2017, the Department of Respiratory Diseases in Shenzhen Children’s Hospital received a cohort of pediatric patients with persistent fever, wheezing and coughing for at least 7 days. Blood tests were conducted immediately after hospitalization to assess the total numbers of leukocytes, neutrophils and lymphocytes counts, as important indicators of infection and inflammation and to measure the concentration of C-reactive protein (CRP) and procalcitonin (PCT), the standard inflammatory markers. All patients were diagnosed using X-ray or Computed Tomography (CT) scanning to measure abnormalities associated with pneumonia. Subsequently, oropharyngeal swabs (155C, COPAN, Murrieta, California, USA) were collected to identify the common pathogens, as mentioned below. Bronchoscopy was required for the disease condition of these patients, allowing the collection of BAL fluid for further pathogen identification by mNGS analysis and for standardized assays to facilitate the clinical decision for treatment, as described below. Negative results for common pathogens and positive for *Mycoplasma pneumoniae* in oropharyngeal (OP) swabs of 32 hospitalized children with severe pneumonia were recruited in this study. Clinical and demographic data of the patients enrolled in this study were collected from the Shenzhen Children’s Hospital electronic patient dossiers. This study was approved by the Ethical Committee of Shenzhen Children’s Hospital with registration number 2016013. Patients were only enrolled after the parents and/or caregivers of included children completed the informed consents.

### Clinical diagnostics using BAL fluid samples

D3 Ultra Direct Immunofluorescence Assay (DFA) Respiratory Virus Screening and Identification Kit (Diagnostic hybrids, Inc, Athens, Ohio, USA) was employed to detect respiratory syncytial virus, adenovirus, influenza virus and parainfluenza virus according to the manufacturer’s instructions. *Mycoplasma pneumoniae* was diagnosed by the Diagnostic Kit for *M*. *pneumoniae* DNA (PCR Fluoresence Probing) (DaAnGene, Guangzhou, China). Bacterial culturing was conducted on blood and chocolate agar plates (Detgerm, Guangzhou, China) to detect *Acinetobacter baumannii*, *Haemophilus influenzae*, *Haemophilus parainfluenzae*, *Moraxella catarrhalis*, *Staphylococcus aureus*, *Staphylococcus haemolyticus and Streptococcus pneumoniae*. The cultured and purified bacteria were identified with matrix-assisted laser desorption ionization-time of flight mass spectrometry (MALDI-TOF) (VITEK MS, bioMerieux, Marcy l'Etoile, France).

### Sequencing and data analysis

In mNGS analysis, mNGS kit for bacteria and DNA viruses was used. DNA was extracted directly from 300ul BALF using the TIANamp Micro DNA Kit (DP316, Tiangen Biotech, Beijing, China) in accordance with the manufacturer’s standard protocols. The extracted DNA was fragmented ultrasonically to yield 200-300bp fragments. DNA libraries were constructed through end-repaired adapter, and by applying polymerase chain reaction amplification to the extracted DNA. Agilent 2100 was used for quality control of the DNA libraries, which were sequenced on BGISEQ-100 platform [8]. All sequencing reagents (PMSEQ) and PMseq software were approved by the China Drug Administration (CDA). All sequencing data were deposited in the GenBank database under accession number PRJNA572371. Processed by removing low-quality and short (length < 35 bp) reads, sequencing data were then aligned with the human reference genome (hg19) to remove human-derived sequences using Burrows-Wheeler Alignment. The remaining data were classified by simultaneously aligning to four Microbial Genome Databases, including viruses, bacteria, fungi, and parasites. The databases were downloaded from NCBI (ftp://ftp.ncbi.nlm.nih.gov/genomes/). It contains 4152 whole genome sequence of viral taxa, 3446 bacterial genomes or scaffolds, 206 fungi related to human infection, and 140 parasites associated with human diseases. The number of unique alignment reads was calculated and standardized to get the number of reads stringently mapped to pathogen species (SDSMRN) and the number of reads stringently mapped to pathogen genus (SDSMRNG) [8]. In case it was regarded as likely causative pathogen, Pathogen-specific sequence was further verified by Sanger sequencing.

### Interpretation of metagenomic data

The microbial list obtained from the analysis process as described above, was compared to the background microbial database, an in-house database which contains microorganisms appearing in more than 50% of the samples in our laboratory in the past three months. Suspected background microorganisms were removed from the microbial list. This was determined based on the unique reads. Microorganisms with SDSMRN <50 unique reads but appearing at least 5 times or SDSMRN of >50 unique reads and appearing at least 3 times were considered as pathogens. For different types of microbes, the thresholds were set as follows:

Bacterial/mycoplasma/chlamydia: SDSMRNG ≥3, if SDSMRN≥3, species was reported; otherwise, the genus was reported
DNA Virus/fungus: SDSMRN ≥3
RNA Virus: SDSMRN ≥1
Parasite: SDSMRN ≥100
*Mycobacterium tuberculosis* complex (MTC): SDSMRNG ≥1

Bacteria were listed in descending order of SDSMRNG, while viruses/fungi/parasites were listed in descending order of SDSMRN. The top 5 species were considered to be significant, and the top 10 for respiratory tract samples of bacterial species. The results were provided to the clinician in an official report and the clinician was required to identify pathogens based on the patient's clinical symptoms.

## Results

### Clinical diagnosis and treatment

The 32 cases were diagnosed with severe nonresponding pneumonia based on diffused lung consolidation, as well as atelectasis or pleural effusion with radiological evidence (including 9 cases with x-ray and 23 with CT-scan). Of these patients, aged from 5 months to 8 years and 7 months, the median age was 21.5 months, 18 were males and 14 were females. The median duration of fever upon admission was 7 days (range 3–30 days), 14 patients had fever for >7 days prior to hospital presentation. Total leukocyte counts ranged from 2.34–21.9×10^9^/L, while total number of lymphocyte and neutrophil counted were 0.58–10.6 and 1.17–22.1×10^9^/L, respectively. Normal values for total leukocytes, lymphocytes and neutrophils were found in 18, 20 and 23 cases, respectively (Table 1). The concentration of serum CRP was higher than 10.0mg/L in 18 cases, and for PCT, higher than 0.5μg/L in 22 cases (Table 1). Empirical antibiotic treatment was applied in all cases and bronchoscopy was used for treatment or diagnostic purposes. Bronchoscopy was performed at a median time of 9 days (range 2–24 days) after admission. Steroid therapy was applied in 20 cases, based on the patients’ conditions.

**Table 1:** Results of laboratory tests on peripheral blood samples derived from 32 pediatric pneumonia patients.

	Normal	High	Low
	(5.0–12.0 × 10^9^/L)	(>12.0 × 10^9^/L)	(<5.0× 10^9^/L)
**Total leukocyte count**	18	7	7
**Lymphocytes**	23	5	4
**Neutrophils**	20	9	3
	**Moderate**	**High**	**No**
	**(**<10 mg/L**)**	**(>**10 mg/L**)**	
**CRP**	14	18	0
**PCT**	**Moderate**	**High**	**No**
	**(**<0,5μg/L**)**	**(**>0,5μg/L**)**	
	10	22	0

### Pathogen detection using oropharyngeal swab and BALF samples

Oropharyngeal (OP) swabs were taken from 32 hospitalized children with severe pneumonia, of these swabs 27 were negative while 5 of the samples were positive for the common pathogens (Table 2), as determined by routine laboratory tests, including bacterial culture, *Mycoplasma pneumoniae* PCR and D3 Ultra DFA Respiratory Virus Screening and Identification. The same routine laboratory tests (culture, PCR and DFA) were also used for pathogen identification in BALF samples, the same pathogens were found as in the OP swabs. In 6 patients, we measured *Mycoplasma pneumoniae* in the BALF samples by PCR. In five of these cases, we also measured *Mycoplasma pneumoniae* in the OP samples, however, the load of *M*. *pneumoniae* was 100 times more in the BALF samples than in the OP samples. In 9 cases, adenovirus was detected by DFA, and in 5 cases, different bacterial pathogens were cultivated (2 cases with *S*. *pneumoniae*, 2 cases with *H*. *influenzae* and 1 with *M*. *catarrhalis*). In 3 cases, co-infection was found, including 2 cases with adenovirus and *M*. *pneumoniae*, 1 case with adenovirus and *H*. *influenzae*. In 15 cases, none of the common pathogens was found in BALF samples (Table 2).

**Table 2:** Pathogens detected in 32 BALF samples using standard clinical diagnostics and mNGS (n.d. = not detected, culture = bacterial culture).

	DFA	PCR	Culture	mNGS
**Adenovirus**	9			25
***Mycoplasma pneumoniae***		6		5
***Streptococcus pneumoniae***			2	2
***Haemophilus influenzae***			2	1
***Moraxella catarrhalis***			1	1
**Cytomegalovirus**				1
**Bocavirus**				1
**Co-infection**	3	2	1	4

### mNGS results in BALF of children with severe pneumonia

BALF specimens were directly sent to the lab for nucleic acid extraction within 2 hours after collection. The approximate time needed for sequencing and data analysis is 18 hours, which is including library construction (8 hours), sequencing (5 hours), data analysis and report interpretation (5 hours). Within 24 hours after collection of the BALF samples, we received the mNGS results, which indicated infection of adenovirus in 25 cases, *Mycoplasma pneumoniae* in 5 cases, *S*. *pneumoniae* in 2 cases, *H*. *influenzae*, *M*. *catarrhalis*, cytomegalovirus (CMV) or bocavirus each in 1 case. In 4 cases, we found a co-infection including 3 cases co-infected with adenovirus and *M*. *pneumoniae* and 1 case with bocavirus and *H*. *influenzae* (Tables 2 and 3). In particular, after receiving the patient result of CMV, we confirmed that the patient was indeed infected by CMV using real time PCR (PCR Fluorescence Probing) (DaAnGene, Guangzhou, China) in the clinical lab. Bocavirus has not been confirmed by qPCR in the clinical lab.

**Table 3:** Pathogen detection based on >50 unique reads of mNGS in 4 co-infection cases. Depth: the ratio of the total amount of the microbial base detected to the detected length of the microbial genome sequence, one of the indicators for evaluating the amount of sequencing.

Patient ID	Depth	Pathogens	Unique reads
**Case 1**	69.87	Human adenovirus B	17653
	1	*Mycoplasma pneumoniae*	146
	14.97	Human adenovirus E	105
**Case 2**	105.44	Human adenovirus B	25230
	20.61	Human adenovirus E	152
	1	*Mycoplasma pneumoniae*	65
**Case 3**	27	Human adenovirus B	7339
	1.9	*Mycoplasma pneumoniae*	7337
**Case 4**	43.54	Human bocavirus	1716
	1	*Moraxella catarrhalis*	810

### Unique reads of mNGS used to distinguish co-infecting pathogens

In patients co-infected with multiple pathogens, we distinguished the main pathogens according to the number of unique reads by mNGS. In this study, we identified 2 patients co-infected with adenovirus and *M*. *pneumoniae* (adenovirus/*M*. *pneumoniae* unique reads 17653/146, 25230/65, respectively) and two other co-infection cases (adenovirus/*M*. *pneumoniae* unique reads 7339/7337, bocavirus/*M*. *catarrhalis* unireads 1716/810) (Table 3).

### Facilitating the optimization of clinical intervention based on mNGS results

After receiving the mNGS results, the antibiotics administration was stopped in the cases where only viruses were identified. Steroid treatment was continued in 20 cases with adenovirus detection. The patient with CMV detected by mNGS was diagnosed with leukemia. After CMV was detected, the treatment was switched from antibiotic to antiviral drug (Ganciclovir) treatment, upon which fever and shortness of breath quickly improved. All 32 patients with pneumonia recovered after 5–45 hospitalization days.

## Discussion

Here we present a proof of principle study using clinical samples obtained from pediatric patients with severe nonresponding pneumonia. The use of mNGS broadens the range of pathogen detection. We have shown the feasibility of a turnaround time of 24 hours, which enables guiding and re-direction of the initial treatment, potentially accelerating the recovery and improving the outcome of disease.

Metagenomic NGS has been used in upper respiratory samples and has been compared to PCR [11–12], however only a few studies have been conducted with BALF samples of CF patients [13]. In this study, we selected 32 hospitalized children with severe pneumonia, in 27 of these cases routine laboratory tests showed negative results for common pathogens in oropharyngeal (OP) swabs and 5 cases were included that were PCR positive for *Mycoplasma pneumoniae*, as a positive control. Limitations of the current diagnostic practice is the delay in results to be able to take the right medical decision [14]. In addition, there is a higher proportion of pneumonia cases with unknown microbial etiology [15], leading to an increase in mortality and to empirical treatment with antibiotics. In the present study, we used OP instead of NP swabs. The OP swab specimen has been found to be superior to the nasopharyngeal (NP) swab specimen for the detection of *Mycoplasma pneumoniae* and adenovirus in children with lower respiratory tract infection, as described by Kim *et al*. and Kakuya *et al*. [16–17]. A total of 32 BALF samples were used for mNGS-based pathogen detection. Six BALF samples were positive for *M*. *pneumoniae* by qPCR, including the 5 positive controls, and in 2 of the 5 positive control BALF samples, adenovirus was detected by the direct fluorescent antigen test (DFA), strongly indicating adenovirus-*M*. *pneumoniae* co-infection. All 32 BALF samples detected pathogens by mNGS. With standard molecular diagnostics, *M*. *pneumoniae* was detected in BALF samples of 6 patients by qPCR. Five of the 6 PCR-positive samples were also positive with mNGS.Thus, sensitivity of the mNGS approach was at least similar to the sensitivity of the diagnostic PCR in this set of samples. The sensitivity of mNGS was higher than that of DFA used for the detection of adenovirus, as only 9 were detected with DFA, while additional 16 were found with mNGS. This results indicate the importance of the use of a nucleic acid-based test for routine diagnosis of adenovirus infection. Adenovirus is an important respiratory pathogen, and the positive rate (78.13%) in our study was significantly higher than that (13.3%) in Hunan[18], which may be related to the number of specimens and selected patients in our study. Furthermore, culture and mNGS were equally sensitive, as only one case of *H*. *influenzae* was missed by mNGS. This may be due to misinterpretation of culture results (i.e. false positive result). In addition, other pathogens were detected that were not found by standard diagnostic tests, such as CMV and bocavirus. The patient in which CMV was detected was not just colonized but infected with CMV, as this patient was diagnosed with leukemia and antibiotic treatment was ineffective, while after switching to antiviral drug (Ganciclovir) treatment, fever and shortness of breath quickly improved. This strongly indicated that CMV was not an innocent bystander, but likely the causative agent of infection. Most striking were the co-infections found in 4 of the 32 patients, in which the added value of mNGS became most clear, as by using only one diagnostic method, co-infection will be missed.

However, this study has a number of limitations. Firstly, only a few pathogens were detected because of the small sample size. Almost in all adenovirus positive cases, multiple adenovirus types were detected (adenovirus B and adenovirus E). This is likely artefact due to sequence homology (similarity). According to our previous studies, most of the adenovirus infections in children are mono infections [19]. The similarity between these sequences (E/B <1%), and the fact that the E-type strain genome sequence is the reference sequence in the alignment library has likely caused this artefact. The time at which BALF the samples are taken (median time 9 days after admission) and after the start of antibiotic treatment (with or without additional steroid treatment), may decrease the detection of bacteria. Finally, we performed only metagenomic NGS to detect bacteria and DNA viruses, not RNA viruses. It is possible that some RNA viruses have not been detected.

The costs of mNGS might still be an obstacle for most hospitals to implement mNGS for routine diagnosis. In addition, efforts and investments are needed to further standardize and validate this method, especially with respect to sample preparation and data processing. Nevertheless, implementation of mNGS in clinical diagnostics is promising and should be embraced by clinicians, primarily because, in some cases, a pathogen cannot be found by conventional molecular methods, in those cases mNGS is superior as a diagnostic tool. Thus far, the limitation was the time mNGS was taken and the difficulty of mNGS data interpretation (for which complicated bioinformatic expertise was needed). In this study, we have shown that those hurdles have now been taken and that the methods are rapid and comprehensible.

## Conclusions

Metagenomic NGS can increase the sensitivity of detection of the causative pathogens in children with severe nonresponding pneumonia. In addition, mNGS will give more strain specific information, will help to identify new pathogens and could potentially help to trace and control outbreaks.
